# Characterizing infection prevention programs and urinary tract infection prevention practices in nursing homes: A mixed-methods study

**DOI:** 10.1017/ice.2023.127

**Published:** 2024-01

**Authors:** Karen M. Jones, Sarah L. Krein, Julia Mantey, Molly Harrod, Lona Mody

**Affiliations:** 1 Division of Geriatric and Palliative Medicine, Department of Internal Medicine, University of Michigan Medical School, Ann Arbor, Michigan; 2 Center for Clinical Management Research, Veterans’ Affairs Ann Arbor Healthcare System, Ann Arbor, Michigan; 3 Division of General Medicine, Department of Internal Medicine, University of Michigan Medical School, Ann Arbor, Michigan; 4 Geriatrics Research Education and Clinical Center (GRECC), Veterans’ Affairs Ann Arbor Healthcare System, Ann Arbor, Michigan

## Abstract

**Objective:**

US policies require robust nursing home (NH) infection prevention and control (IPC) programs to ensure safe care. We assessed IPC resources and practices related to catheter and non–catheter-associated urinary tract infection (CAUTI and UTI) prevention among NHs.

**Methods:**

We conducted a mixed-methods study from April 2018 through November 2019. Quantitative surveys assessed NH IPC program resources, practices, and communication during resident transfer. Semistructured qualitative interviews focused on IPC programs, CAUTI and UTI prevention practices, and resident transfer processes. Using a matrix as an analytic tool, findings from the quantitative survey data were combined with the qualitative data in the form of a joint display.

**Results:**

Representatives from 51 NHs completed surveys; interviews were conducted with 13 participants from 7 NHs. Infection preventionists (IPs) had limited experience and/or additional roles, and in 36.7% of NHs, IPs had no specific IPC training. IP turnover was often mentioned during interviews. Most facilities were aware of their CAUTI and UTI rates and reported using prevention practices, such as hydration (85.7%) or nurse-initiated catheter discontinuation (65.3%). Qualitative interviewees confirmed use of these practices and expressed additional concerns about overuse of urine testing and antibiotics. Although transfer sheets were used by 84% to communicate about infections, the information received was described as suboptimal.

**Conclusions:**

NHs identified IP challenges related to turnover, limited education, and serving multiple roles. However, most NHs reported awareness of their CAUTI and UTI rates as well as their use of prevention practices. Importantly, we identified opportunities to enhance communication between NHs and hospitals to improve resident care and safety.

Robust infection prevention and control is critical to the safe care of nursing home (NH) residents. US payment regulations require NHs to have an infection prevention and control (IPC) program that includes “a system for preventing, identifying, reporting, investigating, and controlling infections” and written policies, standards, and program procedures.^1^ A designated infection preventionist (IP) responsible for this program is also required.^1^ However, meeting these requirements has proven difficult for many NHs, due in part to resource constraints and staff turnover.^2^

To bolster NH IPC programs and reduce healthcare-associated infections, we implemented a 12-month program, Preventing Resistance and Infections by Integrating Systems in Michigan (PRIISM), to enhance relationships between NHs and hospitals.^3^ PRIISM, which began January 2018, brings NH IPC programs and their affiliated acute-care hospital IPC programs together aligning prevention initiatives and provider training across the healthcare continuum. The overarching goals of PRIISM were to reduce infections and enhance resident safety, with specific focus on reducing catheter-associated and non–catheter-associated urinary tract infections (CAUTIs and UTIs). PRIISM was initially developed, tested, and refined in 13 NHs in southeastern Michigan and their 3 main referring hospitals. In the 3 years since it began, an additional 67 Michigan NHs have implemented PRIISM.

The purpose of this evaluation study, which focused on 56 NHs participating during the first 2 years, was to understand key contextual factors among NHs participating in PRIISM at program outset. This analysis included NH IPC program characteristics, IP and staff training, CAUTI and UTI prevention strategies, and communication surrounding resident transfers with hospitals. This information was used to modify program content, to identify specific needs, and to inform ongoing efforts to support NHs meeting regulatory requirements and ensuring safe resident care.

## Methods

### Study design

In this mixed-methods study, we integrated data from cross-sectional NH surveys and semistructured interviews conducted with PRIISM participants during the first 2 years of the program, cohort 1 (ie, the pilot year) and cohort 2. This research was deemed exempt from approval by the University of Michigan Medical School Institutional Review Board.

### Sampling and recruitment

Participating PRIISM NHs were recruited following a recommendation from a local participating hospital or request to participate after learning about the project at a hospital-led postacute care meeting. There were no specific enrollment criteria beyond the expectation that NHs would remain engaged throughout the 12-month participation period. At the beginning of each project year (2018 and 2019), participating NHs received a study survey for completion by the NH administrator, director of nursing (DON), or IP. During these project years, we recruited clinical leadership and PRIISM NH participants, primarily DONs, assistant directors of nursing (ADONs), and IPs to participate in qualitative semistructured interviews. Potential participants from each NH were emailed twice. For those who agreed, an interview was scheduled at their convenience. No response was considered a passive decline.

### Data collection

#### Quantitative data collection

The survey, distributed between January 2018 and March 2019, comprised 36 questions. Survey domains included facility characteristics; IPC program, including experience and training of the individual responsible for IPC and antibiotic stewardship; surveillance activities; CAUTI/UTI prevention practices; and communication with other healthcare facilities.

#### Qualitative data collection

Semistructured interviews with NH staff were conducted between April 2018 and November 2019, while the NHs were participating in the PRIISM program. Interview topics included the same survey domains to gather more contextualized information across NHs (Appendix A online). The questions also focused on experiences with the PRIISM program, relationships with local hospitals, resident transfer between hospitals and NHs, and barriers to and facilitators of implementing IPC practices. Prior to the interview, verbal consent was obtained. Interviews were conducted in person, before or after a PRIISM meeting, or by telephone, and interviews were audio-recorded and transcribed verbatim.

### Data analysis

#### Quantitative analysis

Responses for each survey question were summarized using Stata version 15 software (StataCorp, College Station, TX). Categorical responses were summarized as percentages, excluding any missing responses. Numeric responses were summarized as mean (range).

#### Qualitative analysis

A descriptive matrix analysis approach was used to analyze patterns of responses among participants.^4^ This approach involves creating a table (ie, rows and columns) to analyze domains of interest across sites and/or participants. Within each cell, participant data are synthesized, but they reflect participant perspectives and often contain direct quotes supporting the interpretation. This approach is valuable in identifying congruence and incongruence across domains and participant experience. For this study, the matrix was constructed with rows representing interview topics and columns representing participant roles and NH affiliation. Transcripts were read by 2 study team members (S.L.K. and M.H.), and the responses were summarized and added to the matrix along with applicable supporting quotes. The full study team reviewed the matrix, and if necessary for clarification, transcripts were revisited for additional information to ensure accuracy of interpretation. Each domain was reviewed across participants and was summarized to develop the overall findings. Findings were confirmed through discussion among the study team.

#### Data integration

Using the matrix as an analytic tool, quantitative survey data findings were combined with the qualitative data in the form of a joint display.^5^ This type of integration facilitated development of further insights as qualitative results helped explain and provided additional context to the quantitative findings. Our analysis focused on 3 specific areas to improve understanding of (1) IPC infrastructure; (2) UTI and CAUTI outcomes, process measures, and prevention strategies; and (3) communication with hospitals.

## Results

Surveys were completed by >90% of participating NHs in cohorts 1 and 2. Of 51 NHs, 45 (88.2%) identified as for-profit facilities. The mean bed size was 130 (median, 127; range, 24–286), and 11 (22%) of 50 NHs were enrolled in the National Healthcare Safety Network (NHSN) at the start of the study. Semistructured interviews (average length, 53 minutes) were conducted with 13 participants representing 7 different NH providers: cohort 1 comprised 4 NHs and cohort 2 comprised 3 NHs. Most NHs in the PRIISM program were individual facilities, but some were NH providers with multiple participating facilities. Interviewees included facility administrators, DONs, ADONs, IPs, regional managers, and clinical operations staff (Table 1).

**Table 1.** Nursing Home Participation

Mode	Cohort 1 (Pilot Year) (N=13)	Cohort 2 (N=43)
Surveys	January–March 2018 12 of 13 (92%)	January–March 2019 39 of 43 (91%)
Interviews	April 2018–October 2018 NH 1: IP and administrator NH 2: IP and administrator NH 3: IP and DON NH 4: IP and DON	July 2019–November 2019 NH 5: VP of Clinical Services and 2 Regional Clinical Consultants NH 6: IP NH 7: Director of Clinical Operations

Note. NH, nursing home; IP, infection preventionist; Director of nursing (DON); vice president (VP).

### Infection prevention and control infrastructure

Surveyed NHs reported a mean of 1.11 full-time employees (range, 0–3) dedicated to the IPC program (Table 2). A minority (26.0%) of those in the IP role had been in that facility’s role > 5 years, and most (74.0.%) had <5 years of IP experience. Staff in the IP role frequently held other positions within the facility, often staff education and development (60.8%), employee health (25.5%), and serving as DON (27.5%). On average, IPs performed 1.7 activities (range, 1–6) in addition to their IP responsibilities. In 76% facilities, the IP provided IPC-related training to facility staff.

**Table 2.** Infection Prevention and Control Infrastructure

Survey Item	Total (N=51), No. (%)	Exemplary Quotes
How many full-time employees (FTEs) are currently dedicated to your facility’s infection control program?	Mean=1.11 (Range 0–3) 3 missing responses	*There was several* [talking about IP turnover]… *so it wasn’t a new role….. I did it for maybe 2 months before I took back on the assistant director, did it for 2 months, and I did the infection control and education together*. [DON NH 3] *No, she’s new* [noting turnover, when asked if ADON/IP had an infection control background]. *Yep, she’s pretty excited about it, and I gave her the manual; I’m like, “Here, read the manual. Start with that.” And then we’ll dig into each chapter from there, but just read it and have a sense of what I’m talking about, ‘cause it’s so huge.* [DON NH 2]
No. of years [infection preventionist] in that position in this facility		
<1 year	19/50 (38.0)	
≥1 to <5 years	18/50 (36.0)	
5 years or more	13/50 (26.0)	
No. of years with infection prevention experience		
<1 year	10/49 (20.4)	
≥1 to <5 years	20/49 (40.8)	
5 years or more	19/49 (38.8)	
Are any of the activities listed below also performed by the main point of contact for infection prevention–related issues? (Select all that apply.)		*I just hired an Assistant Director of Nursing, so she’s gonna be taking over my infection control. So, I anticipate it to be, being way better because it’ll be one person doing it versus, I’m the DON doing infection control. Infection control is a huge job, so we are not 100% proficient and always up to date on everything, because I just can’t put the time in that needs to happen*. [DON NH 2] *If you really want somebody to actually do the whole thing, it should be a full-time job, because mostly our infection preventionist has another job of system development so they do orientation and training and it takes a lot of time so you can’t really focus on rounding, and it’s just difficult for them.* [Director of Clinical Operations NH 7]
Staff education/staff development	31/51 (60.8)	
Director of Nursing (DON)	14/51 (27.5)	
Employee health	13/51 (25.5)	
Resident care	11/51 (21.6)	
Other administrative function	8/51 (15.7)	
Other (eg, resident assessment coordinator, unit manager, staff recruitment)	11/51 (21.6)	
No. of additional activities performed by main point of contact for infection prevention–related issues	Mean=1.7 (Range 1-6)	
Who provides infection prevention–related training to the staff at your facility? (Check one answer.)		*I don’t handle the education part – my coworker does – but it’s just on and on. We’re required to do so many and then, whatever gets brought forth. Like, every month, we have to do something related to infection control. That’s our role. So, at least 1 inservice always has to relate and maybe more. But I try to do that antibiotic stewardship too once a year, so they even understand that there are programs…that a program exists*. [ADON/IP NH 6]
The main point of contact for infection prevention–related activities	38/50 (76.0)	
Education Coordinator	21/50 (42.0)	
Director of Nursing (DON)	13/50 (26.0)	
Other (eg, external consultants, medical director, home office, online training)	8/50 (16.0)	
Has the main point of contact for infection prevention-related issues (your facility IP) received any specific infection prevention training? (Select all that apply.)		*Every DON, staff development, infection control nurse, and a lot of the unit managers have become infection control certified, so that’s…that knowledge right there I think is gonna be very, very helpful in the future especially with the antibiotic stewardship*. [VP Clinical Services NH 5]
State or local training course	16/49 (32.7)	
APIC EPI 101 or 201	5/49 (10.2)	
No specific infection control training	18/49 (36.7)	
Is there a committee in your facility that reviews healthcare-acquired infections (hais)? If yes, indicate the members represented in the committee. (Select all that apply.)	49/51 (96.1)	[Response to question about who was on the committee] *The medical director, the pharmacist, as you said, the administrator, director of nursing, the infection control preventionist, maintenance director, housekeeping, sometimes I do attend a meeting just to, you know, oversee the process. I would like to implement, in the future, not yet the nursing assistant and the nurses on the floor are included in those meetings because of the fact that they are the direct care workers that actually can say, this is what’s actually going on.* [Director of Clinical Operations NH 7]
Unit managers or supervisors	44/49 (89.8)	
Medical director	42/49 (85.7)	
Nursing administrators	42/49 (85.7)	
Environmental services	33/49 (67.4)	
Nursing staff	33/49 (67.4)	
Pharmacy department	24/49 (49.0)	
Quality department	18/49 (36.7)	
Physician staff	10/49 (20.4)	
Other (eg, facility board members, resident/family council members, dietary services, social workers)	10/49 (20.4)	

Among facility IPs, 36.7% had no specific IPC training. Nearly all participating facilities (96.1%) had a committee to review healthcare-acquired infections (HAIs). These committees predominantly included unit managers (89.8%), medical directors (85.7%), and nursing administrators (85.7%).

Our qualitative findings supported the survey results (Table 2). Frequent IP turnover, limited IPC experience, and holding multiple roles were commonly described by interviewees. Educating staff about IPC was generally the responsibility of the IP, but educators, DONs and ADONs were often involved. In addition to limited experience, IPs usually had limited training, although some facilities described actively focusing on IP training. Finally, member composition varied among those having an HAI review committee. Although most committees included nurse managers, physicians, and administrators, some NHs included a broader range of staff, such as pharmacy and environmental services.

### CAUTI- and UTI-specific processes and prevention practices

Most respondents were aware of their facility CAUTI and UTI rates (85.7% and 92.0%, respectively) (Table 3). A majority indicated using an electronic health record system to collect CAUTI and UTI data (88.0%), yet most respondents (90.0%) also reported reviewing provider notes to determine resident UTI diagnosis. All respondents shared infection data with NH leadership, 72.0% shared infection data with bedside nursing staff, but only 32.0% shared infection data with residents and families.

**Table 3.** CAUTI/UTI: Outcomes, Process Measures and Prevention Strategies

Survey Item	Total (N=51), No. (%)	Exemplary Quotes
**Aware of CAUTI and UTI rates**		
CAUTI rates	42/49 (85.7)	
UTI rates	46/50 (92.0)	
**Data collection process**		
Collects UTI data, using an electronic health records system	44/50 (88.0)	*I run a report that’s like an order listing report, and I only do it twice a week. I have 14 different classifications that I run in the report. Then I look at that, look the patient up in the system, make sure that do we meet McGeer’s criteria, what happened? If they don’t, I literally call the nurse and say “hey, I saw that this person you put in an order for Cipro, I don’t see any signs or symptoms. Can you tell me what happened with this patient, what’s going on*? [DON NH 2] *I do it by hand. Right. I don’t have a program … I’m able to see every order that’s in the system. I go back and I look, and I see who’s having a UA, who has a Foley, and I keep track of those by hand*. [IP NH 3]
Uses standard definitions to determine whether a resident has UTI (McGeer criteria or CDC NHSN definitions)	49/49 (100.0)	
Uses new antibiotic prescriptions to determine whether a resident has a UTI	22/45 (48.9)	
Reviews provider notes to determine whether a resident has a UTI	45/50 (89.8)	
**Share infection data with:**		*We do feed it back with the staff, but I wouldn’t say we’re the best at sharing with it because we kind of get caught up in the hustle and bustle of day to day*. [Administrator NH 2]
NH leadership	50/50 (100.0)	
Bedside nursing staff	36/50 (72.0)	
Residents and families	16/50 (32.0)	
**CAUTI and UTI prevention strategies**		*I’d say we’re always looking at the patients with Foleys and making sure and discontinuing them if the doctor feels that they’re not necessary. We do education on peri care and how to properly clean the patient. We do lots of hydration, like we pass waters on all 3 shifts*. [Administrator NH 2] DON: *Yes, we have the—well, it’s in connection with prevention—the cranberry protocol*. IP: *Right, because they were given … they were giving the cranberry tabs and UTI stat* [Cranberry Concentrate with added nutrients for urinary tract health], *to everybody who had a urinary tract infection, but it’s not for that. We did just DC—it’s been about a year now—5 long-term people who was on the UTI stat, the cranberry supplements for over a year. I’m tracking them with Dr. [name] to see if they had developed another UTI since the date that they discontinued it and they haven’t, so*. [DON/IP NH 3]
Hydration practices	42/49 (85.7)	
Nurse-initiated indwelling urinary catheter discontinuation	32/49 (65.3)	
Cranberry juice/tablet	31/49 (63.3%)	
Stop orders for indwelling catheters	24/49 (49.0%)	
Multidisciplinary rounds examining indwelling devices	18/49 (36.7%)	
Electronic alerts and reminders of indwelling catheter need	13/49 (26.5%)	
Other (eg, hand hygiene, urology visits, audits)	11/49 (22.5%)	
No prevention strategy	2/49 (4.1%)	

UTI and CAUTI prevention strategies included hydration practices (85.7%), nurse-initiated indwelling urinary catheter discontinuation (65.3%), stop orders (49.0%), indwelling device rounds (36.7%), and electronic alerts or reminders to assess indwelling catheter need (26.5%). Nearly two-thirds (63.3%) of NHs reported using cranberry juice and/or tablets as a UTI prevention strategy. Two facilities (4.1%) reported no prevention strategies.

Consistent with quantitative findings, our qualitative results revealed various strategies to collect CAUTI and UTI data, but in general most facilities used a manual data extraction process. This process included reviewing provider notes, laboratory data, antibiotic prescription information and managing the information in spreadsheets. Most interviewees used Revised McGeer Criteria to identify resident infections. Sharing data with floor staff varied across NHs. Data were shared most often in the context of education and practice change. One interviewee stated that although data was shared with floor staff, they felt there was not enough time to discuss what it meant and how to improve.

To prevent CAUTIs, most NHs indicated providers were amenable to discontinuing indwelling urinary catheters on or soon after admission. This was attributed to previous education around prompt catheter discontinuation and catheter assessments at admission. In some NHs, nurse managers assessed catheter appropriateness. To prevent CAUTI and UTI, all NHs mentioned hydration protocols, and at least 2 described using “cranberry protocols,” which included giving high UTI-risk residents UTI-Stat, a cranberry concentrate.

Another area of focus involved residents admitted with or placed on antibiotics for suspected UTI, without symptoms or urine-culture results. Several interviewees stated educating staff nurses about symptoms and urine testing appropriateness was an ongoing task. To address this issue, one NH implemented testing with a urine dipstick before calling the physician for a urine culture order. Another NH implemented a UTI-focused assessment process or Situation-Background–Assessment–Recommendation (SBAR). As the IP explained,

“*It’s just specific for UTIs. It goes over in the first section, the basic, is there a fever, does he have any flank pain, any acute pain, any shaking or chills, any mental status changes even though that’s not a clinical sign of a UTI, but if we have these checked for the patient, then we’ll go on. Is there urgency, is there frequency, is there suprapubic pain, hematuria? These are just stuff that the nurse’s looking at so they could have a clear picture when they call the doctor*.” [Infection preventionist, NH 3]

### Infection-related communication with hospitals

NHs shared information with other healthcare facilities about infections and MDRO history during resident transfers through a variety of mediums. When transferring residents in or out of the facility, infections were communicated using transfer sheets (83.7%), discharge orders (65.3%), phone calls (38.8%), and uniform assessment instruments such as SBAR (12.2%) (Table 4). MDRO histories of residents were communicated via transfer sheets (85.4%), discharge orders (56.3%), phone calls (39.6%), and uniform assessment instruments (10.4%).

**Table 4.** Infection-Related Communication with Hospitals

Survey Item	Total (N=51), No. (%)	Exemplary Quotes
How are infections (any) communicated when transferring residents in and out of your facility? (Select all that apply.)		*I don’t get the microbiology results, I just get a certain amount of paperwork in there, and we have our processes. I get emailed on every admission. … Nurse on the floor that’s discharging the patient usually calls, just to give handoff. And it’s not always effective 100% either. I know sometimes you have nurses have said “Hey, we tried calling. No one’s answering the phone*.” [DON NH 2] *Some of the other facilities, they won’t even tell you what it is they have. Just like the CRE, they never told us … they [admissions coordinator] give us what they have reviewed because they’re not nurses. They’re not medical, and then a lot of time the information is uploaded into the system already when they say, hey, we’re getting this admission. That’s how we went in and saw that the person had CRE*. [DON/IP NH 3] … *and then just regular admissions, it takes forever. It’s a lot of time to read through 100-page transfer… packets to try and find the culture or why they’re on the you know… It’s a lot of work. … your average floor nurse on a Saturday is not looking for any of that. And if somebody was to be on precautions, I can guarantee you 90% of the time it’s not gonna happen, so it’ll be Monday and by that time, you’ve already exposed you know, however many people*. [Clinical consultant NH 5]
Transfer sheet	41/49 (83.7)	
Discharge orders	32/49 (65.3)	
Phone call	19/49 (37.5)	
Uniform assessment instrument (eg, SBAR)	6/49 (12.2)	
Other	4/49 (8.2)	
How are patients with a history of MDROs communicated when transferring residents in and out of your facility? (Select all that apply.)		*The difficulty is the fact that if we have a patient that is going to be sent to the hospital from here, it’s so hard to get in touch with the emergency department. And then our end, also, … with such a huge building like this, phone calls go to another unit, and they think like, okay, nobody’s answering the phone and all that stuff.* [Director of Clinical Operations NH 7] *I really think that a lot of hospitals and maybe it’s not even a lot, but I’m gonna say the word a lot, a lot of hospitals, as soon as you walk through the door, you have a UTI and you’re on antibiotics… And I think a lot of times the hospitals treat our residents that are already colonized*. [VP of clinical services NH 5]
Transfer sheet	41/48 (85.4)	
Discharge orders	27/48 (56.3)	
Phone call	19/48 (39.6)	
Uniform assessment instrument	5/48 (10.4)	
Other	2/48 (4.2)	

Although the survey results suggested what appeared to be a relatively standard process for communicating infection-related information, our qualitative findings provided a different picture, highlighting several communication challenges. For example, NHs received either too little or too much information, making it difficult to get a timely and complete picture of the resident’s status. In addition, most interviewees described challenges related to those involved in the communication process, with admission information being sent to an admissions person who may or may not have a clinical background and little to no direct clinician-to-clinician communication between the hospital and NH. Finally, from the NH viewpoint, there was a perception that residents admitted to the hospital were assumed to have an infection, ultimately ending up on antibiotics.

## Discussion

Developing and maintaining an effective IPC program is a crucial part of ensuring safe care for NH residents. Our mixed-methods study identified not only several challenges but also positive use of prevention practices and opportunities related to IPC among NHs participating in the PRIISM program. First, IP turnover, filling multiple roles, and having limited IP experience were common challenges. Second, tracking, awareness, and reporting of infection rates (notably CAUTI and UTI) were nearly universal. Most NHs reported using recommended practices to reduce indwelling urinary catheter use and to prevent UTIs, along with some nonbeneficial practices. Finally, although most NHs reported strategies for sharing infection-related information during resident transfers, opportunities for enhancing IPC-related communication between NHs and hospitals still exist.

To receive US federal funding, NHs are required to have a designated IP with specialized IPC training.^1^ Our work highlights the specific challenges of maintaining consistent, appropriately trained IP staff, an issue that is both more important and perhaps more difficult due to the COVID-19 pandemic. At most PRIISM NHs, the IP was in the position <5 years, and more than one-third of NHs reported the IP lacked specific IPC training. Often, IPs were responsible for other activities, such as staff education, employee health, or served as the DON. These findings suggest a focused need for identifying and implementing strategies that help NHs recruit and retain dedicated IP staff, and providing these IPs with the specialized education and knowledge they need. In addition to ensuring IP staff have time and resources needed to attend credible training courses, such as those offered through the CDC and Association for Professionals in Infection Control and Epidemiology (APIC), a coaching and mentoring program might be a useful strategy for providing ongoing education and support.

Recommended practices for preventing CAUTI and UTI in NH residents include general practices (eg, surveillance and hand hygiene) and specific practices (eg, reducing indwelling catheter use) as well as improving diagnosis.^6^ More than 80% of participating facilities indicated that they conduct surveillance, that they are aware of their CAUTI and UTI rates, and that they share these data with NH leadership. Indeed, this group of NHs reported engaging in these activities more often when compared to a cohort of NHs participating in a national CAUTI and UTI–focused collaborative program beginning in 2014–2015, in which only 58% knew their CAUTI rates and 70% shared data with leadership.^7^ Based on the survey and qualitative findings, PRIISM NHs demonstrated a clear focus on strategies to reduce indwelling catheter use, including nurse-initiated discontinuation, and assessment and timely removal if not indicated. Both hydration and use of cranberry products were among the CAUTI and UTI prevention practices reported by most PRIISM NHs. Although evidence to support hydration as a UTI prevention practice is limited,^8^ a recent study of structured drink rounds to promote hydration found a reduction in the average number of UTIs requiring antibiotics (1.8 at baseline to .75 after the intervention) and those requiring hospital admission.^8^ Further research focusing on hydration practices in NHs for UTI prevention is warranted. However, studies of cranberry use continue to demonstrate little benefit among this population and may be a practice suited for de-implementation.^9–11^

Although not included in the survey, antimicrobial use and urine testing for possible infection was a common topic of discussion during the qualitative interviews. In 2017, point-prevalence data indicated that ∼1 in 38 US NH residents received an antibiotic for a UTI on any given day.^12^ This included antibiotics prescribed for UTI prevention, the likely overuse of fluoroquinolones, and median planned duration beyond 7 days for almost all commonly prescribed antibiotic classes.^13^ Concerns about antibiotic overuse and misuse prompted development of consensus recommendations specifically related to diagnosis, treatment, and prevention of UTIs in postacute and long-term care settings by the Infection Advisory Subcommittee of AMDA, the Society for Post-Acute and Long-Term Care Medicine.^13^ With several studies and emerging interventions focusing on promoting appropriate antibiotic use and diagnosis of UTIs in NHs,^13,14^ our findings suggest a high level of awareness and interest among NHs with a desire for strategies to address these issues.

Improving IPC-related communication between NHs and local hospitals was one of the motivating factors for launching PRIISM.^3^ Thus, it is not surprising that our findings identified challenges and areas for improvement in this domain. Although most facilities indicated using transfer sheets, interviews revealed issues with the amount and type of information received, and individuals involved in exchanging information. In general, there appeared to be little inter-clinician communication and the perception that hospitals assumed NH residents sent to the hospital had an infection. Some of these issues are reflected in other studies.^13,15,16^ Nonetheless they highlight the need for strategies and tools to improve interfacility infection-related communication practices. Such strategies include encouraging use of communication tools, such as the CDC’s Inter-Facility Infection Control Transfer Form,^17^ INTERACT,^18^ and consideration of a statewide registry system (eg, the Illinois “XDRO registry”^19^) with additional research focusing on developing strategies that improve NH and hospital communication and coordination.

Linking quantitative survey and qualitative interview data to provide a comprehensive understanding of NH IPC programs and UTI prevention practices is a strength of this work. However, our study has several limitations. Surveys and interviews were conducted with PRIISM participants, a selected group of NHs from a single geographic region, with potential for response bias and limited generalizability. Interviews were also conducted during program participation, which may have affected some responses. Finally, data for this evaluation were collected prior to onset of the COVID-19 pandemic, and our findings do not reflect the potential impact of COVID-19 on NH IPC resources and practices.

In conclusion, IPC is a core element for the safe delivery of NH resident care. Unfortunately, workforce-related challenges, including IP turnover, limited education, and filling multiple roles, were common among NHs enrolled in the PRIISM project. Despite these challenges, participating NHs reported awareness of their CAUTI and UTI rates and use of recommended prevention practices. However, communication of infection-related information during transfers was a clear area with opportunities for improvement and further research.

## Appendix A. Selected Question Domains

**Table:**

Survey (Quantitative) Question	Interview (Qualitative) Question
In your facility, what level of professional training does the main point of contact for infection prevention related issues have?	What is your degree and current role?
How many years of experience does the main point of contact for infection prevention-related issues have in that position at this facility?	How long have you worked at this nursing home?
What activities listed below are also performed by the main point of contact for infection prevention-related issues?	What responsibilities does the Infection Preventionist have?
Is there a committee in your facility that reviews healthcare-acquired infections? Is your facility currently enrolled in the CDC National Healthcare Safety Network? How do you currently monitor infection rates?	Can you please describe your facility’s infection prevention program?
Is surveillance for CAUTI performed at your facility? If yes, where is surveillance data entered?	Does your facility collect data on CAUTIs? Who collects that data? Who do you report that to?
Are any of the following quality improvement (QI) programs for UTI prevention in place at your facility? (Select all that apply)	Can you tell us what initiatives you have in place to reduce UTI?
Please indicate when training is offered for the following topics. These trainings may be provided by facility staff members or external organizations.	What resources do you use to educate your staff about infection prevention/detection/treatment?
How are infections communicated when transferring residents in and out of your facility?	Can you describe for us the intake process? In what form do you receive this information?
